# 

**Published:** 1888-11-24

**Authors:** 


PERMANENTLY ENLARGED TO 32 PAGES.

PRICE ONE PENNY.

No. 113, Vol. V.—November 24th, 1888.

[Registered at the General Post Office as a Newspaper.]

Per Annum, 6s. 6d., post free.
Monthly Parts, 6d.; post free, 7½d.
Ditto per Ann., 7s. 6d. post free.

### THE HOSPITAL

A Weekly Institutional Journal of

